# A term-based and citation network-based search system for COVID-19

**DOI:** 10.1093/jamiaopen/ooab104

**Published:** 2021-12-14

**Authors:** Chrysoula Zerva, Samuel Taylor, Axel J Soto, Nhung T H Nguyen, Sophia Ananiadou

**Affiliations:** 1 Department of Computer Science, National Centre for Text Mining, Manchester Interdisciplinary Biocentre, The University of Manchester, Manchester, UK; 2 Department of Computer Science and Engineering, Universidad Nacional del Sur & Institute for Computer Science and Engineering (ICIC, UNS–CONICET), Bahia Blanca, Argentina; 3 The Alan Turing Institute, London, UK; 4 Chrysoula Zerva's affiliation at the time of submission/publicationis is Instituto de Telecomunicações (IT), Lisbon, Portugal. All work was carried out while the author was employed at the University of Manchester, UK

**Keywords:** term extraction, citation network, exploratory search systems

## Abstract

The COVID-19 pandemic resulted in an unprecedented production of scientific literature spanning several fields. To facilitate navigation of the scientific literature related to various aspects of the pandemic, we developed an exploratory search system. The system is based on automatically identified technical terms, document citations, and their visualization, accelerating identification of relevant documents. It offers a multi-view interactive search and navigation interface, bringing together unsupervised approaches of term extraction and citation analysis. We conducted a user evaluation with domain experts, including epidemiologists, biochemists, medicinal chemists, and medicine students. In general, most users were satisfied with the relevance and speed of the search results. More interestingly, participants mostly agreed on the capacity of the system to enable exploration and discovery of the search space using the graph visualization and filters. The system is updated on a weekly basis and it is publicly available at http://www.nactem.ac.uk/cord/.

## Lay Summary

In this article, we present a search system and exploratory tool built on the documents of the COVID-19 Open Research Dataset, which is a large and open collection of scholarly articles related to COVID-19 (Coronavirus disease 2019), SARS-CoV-2 (Severe Acute Respiratory Syndrome Coronavirus-2), and related coronaviruses. The search system aims to facilitate navigation of the scientific literature related to various aspects of the pandemic. Specifically, we identify 3 types of core information per paper to be used as navigation facets including technical terminologies, citation/reference links from 1 paper to others, and bibliometric data. Unlike other exploratory-based search engines, our system allows users to combine information from text mining and bibliometrics analysis to explore the data in a more versatile manner tailored to their needs. The system is automatically updated on a weekly basis to ensure timely and updated access to recent information. We also conducted a user evaluation that included epidemiologists, biochemists, medicinal chemists, and medicine students. In general, most users were satisfied with the relevance and speed of the search results. More interestingly, participants mostly agreed on the capacity of the system to enable exploration and discovery of the search space using the graph visualization and filters.

## INTRODUCTION

The COVID-19 pandemic resulted in an unprecedented production of scientific literature spanning several fields. Although the primary focus was on the biomedical domain (from virology to vaccines and therapeutics), there were multiple other domains affected, such as socioeconomic studies, politics, etc. Alongside scientists, a broad group of other practitioners wish to consult the continuously changing literature to make informed decisions about patient care, social and work policies, and guidelines. To support navigation through the scientific literature, we developed a search tool that filters information based on technical terms (concepts). The resulting documents are visualized as a connected graph that enables users to visually explore explicit and implicit connections among documents by means of citation information and the cooccurrence of important terms. Our search system is developed based on the CORD-19 Open Research Dataset,^1^ a continuously updated collection of multi-domain, scientific publications relevant to COVID-19, henceforth referred to as *CORD-19*.

## BACKGROUND AND SIGNIFICANCE

We argue that navigating through the rapidly growing COVID-19 literature requires the support of an interactive visual interface that facilitates search and exploration of scientific literature using different facets derived from both text mining and citation analysis. Our system integrates text mining and citation analysis results with visual text analytics.

Several groups responded to the COVID-19 emergency to facilitate literature navigation through the development of search systems. These are divided into 3 main categories: (1) information retrieval (IR) search systems,^2^^,^^3^ (2) question-answering (QA) search systems,^4^^,^^5^ and (3) exploratory search systems (ESSs).^6^^,^^7^ It is noted that many of the surveyed tools take advantage of recent advances in natural language processing based on the application of language models^8^ for search and semantic inference. These language models are deep neural network architectures trained to model language on large unlabeled corpora and then fine-tuned on data that are closer to the target corpus and task. Language models can be trained on multiple languages and can be trained either on text from the generic domain, for example, BERT,^9^ T5,^10^ BART,^11^ and ALBERT,^12^ or be more focused to specific domains such as scientific text, for example, SciBERT,^13^ or biomedical and clinical documents, for example, BioBERT^14^ and BlueBERT.^15^

### IR search systems

IR systems only retrieve related documents, focusing on indexing (*indexing* when used as a term in this article refers to storing structured representations of text in a way that allows to map them to corresponding representations of search queries) them and providing efficient ways of ranking documents by relevance to a given set of queries. CO-Search^2^ employs a pretrained SBERT model^16^ to index paragraphs as well as image captions. Neural Covidex^3^ uses a keyword search component and a reranker to improve ranking quality. The keyword search is built using Pyserini, a Python binding for Anserini,^17^ where documents are ranked based on the relative keyword frequency of a given document when compared with the query as well as the rest of the documents (BM25 algorithm). The output of Pyserini is then reranked by a T5 language model,^10^ which is fine-tuned on MS MARCO, a large machine reading comprehension dataset.^18^ Similarly, SLEDGE^19^ uses a similar approach, but using SciBERT^13^ to rerank documents.

### QA search systems

QA search systems handle user queries as questions providing answers by retrieving and summarizing the relevant snippets from the available documents. CAiRE-COVID^4^ is such a system based on a query-focused multi-document summarization system with a document retriever implemented for paragraph indexing using Anserini.^17^ CAiRE-COVID uses an ensemble of 2 QA models: HLTC-MRQA^20^ and BioBERT.^14^ It fine-tunes BART^11^ and ALBERT^12^ to include both abstractive and extractive summarization.

CovidAsk^5^ allows users to ask questions related to COVID-19 by showing relevant documents with highlighted answers and important entities to a question. SciFact^21^ verifies scientific claims related to COVID-19 by either supporting or refuting a claim based on scientific evidence.

### Exploratory search systems

ESS supports faceted search interfaces (ie, searching and filtering on specific metadata values) and interactive visualizations to narrow down search results in the document collection, instead of just allowing text queries. SciSight^6^ combines search facets and filters using a collocation explorer and a coauthorship network. S2ORC-SCIBERT^7^ has been fine-tuned on 7 biomedical datasets including GENIA^22^ and BC5CDR.^23^

Our proposed system is also an EES but has different functions compared with SciSight and S2ORC-SCIBERT. Specifically, it provides users with the following: (1) term extraction and visualization representing the most important terms in the search results; (2) term and metadata-based search facets to organize and refine retrieved documents; and (3) a document citation network with citation and term cooccurrence links. The terms are extracted in an unsupervised manner providing cross-domain information. Our system offers multifaceted filtering and navigation panels that allow users to combine information from text mining and bibliometrics analysis to support information discovery and explore data in a versatile manner.

## MATERIALS AND METHODS

The CORD-19 dataset is used as our main dataset. We identify 3 types of core information per document to be used as navigation facets:

1. Terms: text spans signifying technical terminology and/or keywords that summarize the main topics of a document and are associated with a corresponding weight of importance within each document. Such terms are used both to filter documents and to identify semantic relations between them
2. Citation links: references to other papers can be used to indicate topical relations between papers, and also facilitate the identification of *authoritative* (multi-cited) documents as well as documents acting as *hubs* (review or meta-analysis publications citing core documents)
3. Bibliometric data: additional information, such as the publication time and venue, can also provide useful filters, reducing search time.

### Indexing

Elasticsearch (https://www.elastic.co/), an open search and analytics engine, is used to index the CORD-19 documents. We initially experimented with different indexing schemes on a subset of the data, comprising 51K documents, which were used for round 1 of the TREC-COVID challenge^24^^,^^25^—a document retrieval challenge where a set of 50 queries is provided and documents relevant to each query are annotated. We compared the indexing performance on different text units: (1) using the full raw text, (2) using only the title and the abstract as raw text, (3) using the full text but indexing each paragraph separately and then mapping back to the document, (4) same process as (3) but using only the first and last sentences of each paragraph, and (5) reranking Elasticsearch results based on the frequency of term cooccurrences between the query and the document, namely term-based reranking.

We considered several IR metrics for our evaluation: normalized Discounted Cumulative Gain (nDCG), mean average precision (MAP), and precision@N for ranks *N* = 5 and *N* = 10. Each metric accounts for different performance properties:

- Precision@N captures how relevant to the query are the top-*N* results returned by the system.
- nDCG is a more robust metric considering several ranking properties: it accounts for a sorting where prioritizing very relevant results over somewhat relevant results is preferred (cumulative gain) and the higher in the rank they appear, the higher the score. Finally, it provides a normalized score so that the value is not dependent on specific queries.
- MAP estimates a combination of performance for precision and recall at the top-*K* rank positions, normalized over a set of queries, where *K* is the number of relevant documents.

We also calculated the running time of 50 random queries (50Qtime) since maintaining time efficiency remains a key aspect for an exploratory search index.

The results in Table  1 indicated effective retrieval performance, especially when using paragraph-level indexing or sentence selection (selecting the first and last sentences of each paragraph). Term-based reranking appeared to be effective although it significantly increased the processing time, which motivated us to a follow-up change for the current version to index the text jointly with automatically extracted terms instead of a *post hoc* reranking. For comparison, we provide the performance across metrics for the top performing system in round 1 of the TREC-COVID challenge,^25^ since we used the round 1 topics to estimate the performance of our system too. It can be seen that our system is competitive especially for the metrics related to precision of top-ranked results. We note that the performance of the systems seems to be highly dependent on the topic as well as on the data (document) pool; we thus also provide the performance of Anserini^17^ used as the round 0 baseline system as reported in the TREC-COVID challenge,^25^ for a better contextualized comparison.

**Table 1.** Performance on the 10APR2020 data (round 1) for different indexing units

	TREC round	nDCG	MAP@10	P@5	P@10	50Q time (min:s)
(OUR) Full text	1	0.391	0.170	0.549	0.433	03:30
(OUR) Abstract + title	1	0.403	0.148	0.620	0.400	**03:03**
(OUR) Paragraph	1	0.582	0.165	0.680	0.648	04:23
(OUR) First + last paragraph sentence	1	0.625	0.155	0.704	0.700	04:35
(OUR) Term-based reranking	1	**0.689**	0.190	0.715	**0.745**	12:56
Sabir (sab20.1.meta.docs)^25^	1	0.608	**0.313**	**0.780**	**—**	**—**
Anserini—title/abstract	0	0.606	0.356	**—**	0.510	**—**
Anserini—paragraph	0	0.503	0.395	**—**	0.503	**—**

*Note*: Bold numbers denote the best results for each metric in round 1. The row in green background highlights the best system in our experiments, and the rows in gray background denote round 0 baselines.

Aiming for a continuously updated and ever-expanding dataset (currently CORD-19 expands by ∼10K documents per week), query execution time poses a significant limitation and maintaining the described functionalities and multiple types of annotations while providing real-time search results is a key desideratum. Considering the heavily nested document structure and the high cardinality of the related terms, we optimized the response time to ∼600 ms on 150K documents by flattening and deduplicating the annotations data structure.

### Term extraction

**Figure 1.**
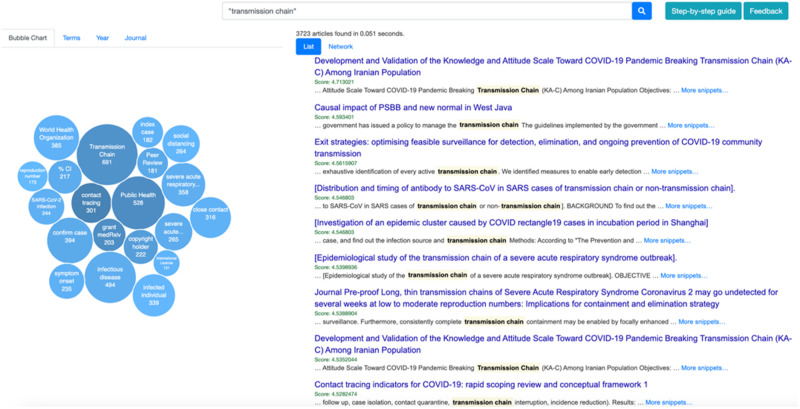
List of results and associated term bubble chart view for the query “transmission chain.” The screenshot was captured on 19 July 2021.

Technical terms were extracted using C-value,^26^ a method that automatically extracts *multi-word terms* and *nested terms*, and ranks them by their importance in a document collection. For example, “noninvasive positive pressure ventilation failure” is a multi-word term that includes nested terms “positive pressure ventilation,” “pressure ventilation,” and “ventilation failure.” The top terms identified by TerMine^26^ are visualized as a bubble word cloud, as illustrated in Figure  1. The most representative terms, that is, those with the highest *C*-value, are represented as *bubbles* with their size being proportional to the *C*-value number. The user can also interact with the bubbles, by clicking on a specific term bubble, to dynamically generate a new search query. The Terms tab also shows the list of terms with their importance (*C*-value) in the document set.

### Document graph construction

**Figure 2.**
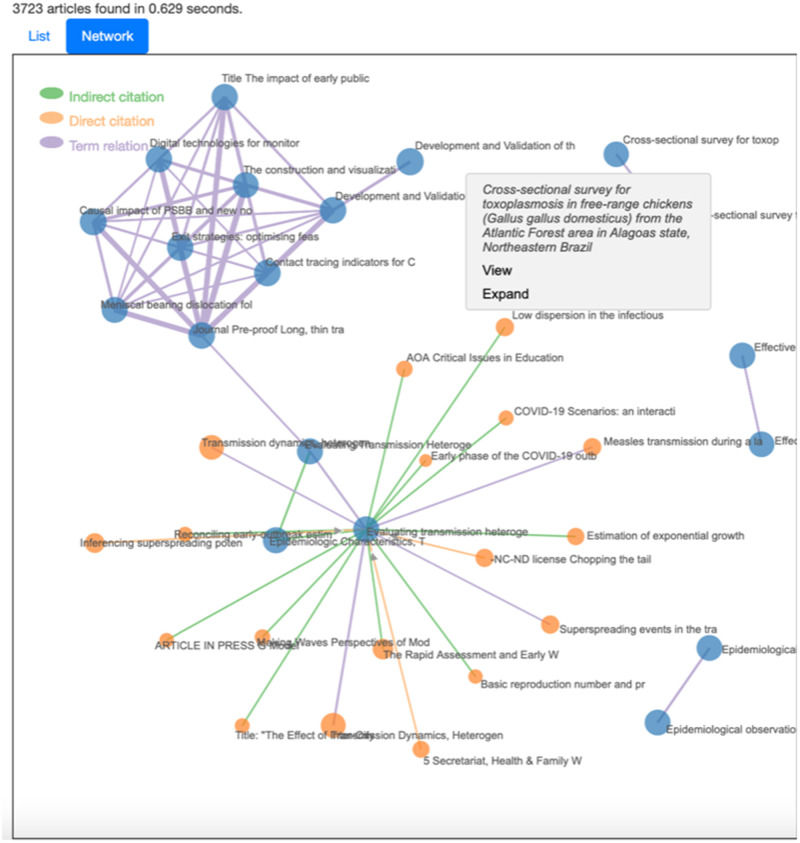
Document graph demonstrating connections between documents returned from the query “transmission chain.” Node size signifies relevance to query. Blue nodes correspond to documents returned within the first 50 results. Orange nodes appear after expanding the blue node in their center. Thicker edges correspond to higher relation weights (see top cluster of purple edges); hovering over an edge will show the weight and direction, and if it is a term edge it will show the cooccurring term. The screenshot was captured using data available on/before 19 July 2021.

Search results are typically presented in the form of an ordered list.^6^^,^^27^^,^^28^ Complementing the standard ranked list, our system adds a document graph view of the results, as shown in Figure  2. This weighted graph allows the visualization of the retrieved documents and their underlying connections. Document graphs can capture and depict richer information compared with document retrieval lists^6^ and consolidate information beyond query relevance, such as bibliometric details, recency information, and interdocument proximity. We opt for a combined depiction of both bibliometric and contextual information by incorporating 2 different types of citation edges (direct and indirect) and term edges. Both edge types use weights signifying the proximity/relevance between document nodes. Specifically, the edges and associated weights are determined as follows:

- **Term edges** signify a relation between documents based on a cooccurring term in both of them. Assuming a term t occurring both in documents a and b, the weight w for the edge $t_{ab}$ (strength of the relation) is calculated as the average *C*-value (term importance) between those 2 terms:
  (1) $$w\left(t_{ab}\right)=\frac{1}{2}\left(C_{\mathrm{value}}\left(t_{a}\right)+C_{\mathrm{value}}\left(t_{b}\right)\right)$$

The edge direction is determined by the relative publication date of each paper and directs from the newest to the oldest publication.

- **Direct citation edges** correspond to a citation mentioned from document a to document b. Edge directionality follows the citation order (from the latest to the oldest paper). The edge weight is defined by a combination of zoning, frequency, and recency criteria. Based on Thelwall^29^ and Nazir et al,^30^ citations in the introduction and related work sections have lower significance with respect to the citing paper. Additionally, the frequency of citing the same paper shows a positive correlation with the relevance of the cited paper. Finally, we introduce a time distance metric to capture the recency of the cited paper w.r.t. the citation. Our assumption is that papers published closer together are more relevant. Combining the above, Equation (2) shows the weight w for a paper a citing paper b, assuming there are N repetitions of the citation ($\mathrm{freq}\left(\mathrm{cit}\left(b\right)\right)=N$), and that each section i has its own citation associated weight ${sw}_{i}$.
  (2) $$w\left({cd}_{ab}\right)=\frac{N\cdot \max_{i=0}^{N}\left({sw}_{i}\right)}{{\max(1,\;\mathrm{year}}_{a}-{\mathrm{year}}_{b})}$$
- **Indirect citation edges** correspond to underlying relatedness between papers, indicated by cocitations, that is, citation cooccurrence. We consider that a paper a and a paper b citing the same set of documents $C=\{c_{1},c_{2},\ldots ,c_{n}\}$ are indirectly related since they refer to the same previous research. The weight of the indirect link is analogous to the combined weight of the common citations C, as shown in Equation (3).
  (3) $$w\left({\mathrm{cid}}_{ab}\right)=\sum_{c\in C}^{}\frac{w\left({cd}_{ac}\right)+w\left({cd}_{bc}\right)}{2\cdot \left|C\right|}$$

This mathematical representation of a graph is visually encoded as an interactive force-based network. The size of each node is proportional to the relevance of the document to the query, while edge widths represent the weight of each connection. Different colors are used for edges to differentiate whether they represent cooccurring terms (purple), direct citations (orange), or cocitations (green).

To make this exploration scalable to varying numbers of search results,^31^ interactive functionalities are implemented for filtering, zooming the graph in and out, and expanding nodes on demand. The filtering allows limiting the number of edges in the graph by setting an edge weight threshold so that a user can focus on the most important connections first. The expansion option enables exploration of salient edges of a single document. The introduced nodes are represented using a different color to differentiate them from the previously visualized nodes. Figure  2 shows a snapshot of the graph for a given query. The interface has been programmed in Javascript and uses the d3.js library for interactive visualization.

### Subject matter expert evaluation

**Figure 3.**
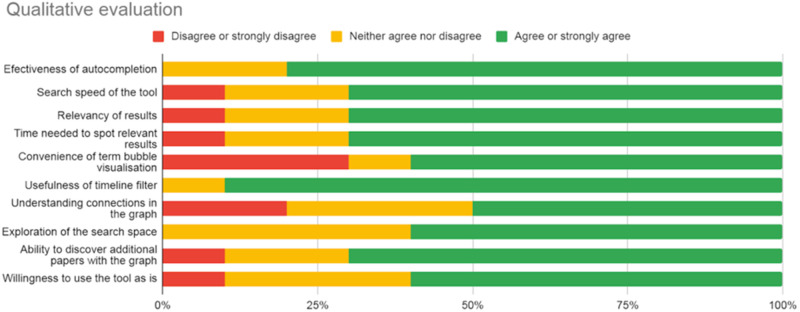
Summary of participants’ responses to different aspects of the tool. Although a 5-Likert scale is used in the questionnaire, a 3-Likert scale is used in the plot for better identification of patterns in the responses.

We conducted a user evaluation with 10 participants, including epidemiologists, biochemists, medicinal chemists, and medicine students. Participants received a 5-minute demonstration and then interacted with the tool using research queries of their own interest. At the end, they completed an online questionnaire. Although the number of individuals is relatively small, an external qualitative evaluation with target expert users quickly helps identify potential issues or obtain valuable feedback on the strengths of a system. A summary of the assessment is depicted in Figure  3. In general, most users were satisfied with the relevance and speed of the search results. More interestingly, participants mostly agreed on the capacity of the system to enable exploration and discovery of the search space using the graph visualization and filters. We noted that some users felt uncomfortable about interacting with the graph and with the complexity of the multiple types of connections in the graph. We are considering allowing users to toggle to show a simplified view of the graph, where all types of edges are aggregated into one and connections of a document can be also explored as a ranked list. We also plan on testing the system in the context of an ongoing public health project that aims at investigating COVID-19 transmission.

## CONCLUSION

The COVID-19 pandemic and its international health emergency have sparked unprecedented mobilization and international collaboration between researchers across different fields. As a result, there has been an exponential increase in the related scientific literature, published both in peer-reviewed and preprint format. To respond to the challenges from navigating through this vast amount of information, we have developed an interactive faceted search system which supports navigation and visualization of the literature. The system through its semantic filtering, and the exploration of explicit and implicit links between retrieved documents, facilitates navigation, and information discovery.

## FUNDING

This work was supported by Biotechnology and Biological Sciences Research Council grant number BB/P025684/1; Lloyd’s Register Foundation, grant: HSE Discovering Safety; and European Commission grant number 874703.

## AUTHOR CONTRIBUTIONS

CZ, AS, and SA were responsible for initial conceptualization of this article. ST did term extraction, document indexing, and developed the search engine. CZ constructed document graphs while AS did the visualization of the search results. CZ, AS, NN, and SA provided feedback on the article, participated substantively in revision, and approved the final version.

## CONFLICT OF INTEREST STATEMENT

None declared.

## DATA AVAILABILITY

The source code is available at https://github.com/nactem/cord.
